# Supplementary material for: Nanoengineering room temperature ferroelectricity into orthorhombic SmMnO3 films
Source: Nat Commun. 2020 May 5;11:2207. doi: 10.1038/s41467-020-16101-2 (PMC7200746; doi:10.1038/s41467-020-16101-2)
Supplement: Supplementary file 1 — Supplementary Information [file 41467_2020_16101_MOESM1_ESM.pdf]

Nanoengineering Room Temperature Ferroelectricity into Orthorhombic $SmMnO_3$ Films, Choi *et al.*, Supplementary Information

Supplementary Discussion

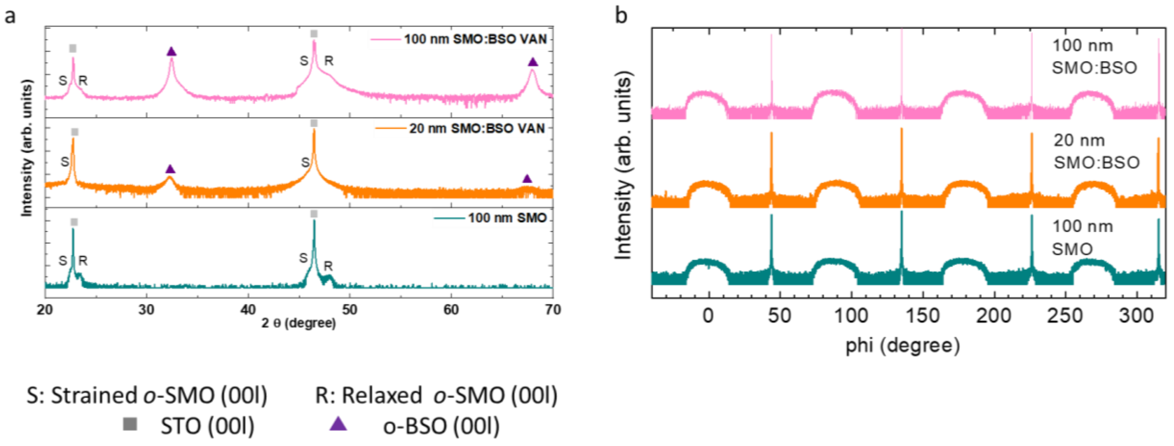

Supplementary Figure 1. Structural analysis by XRD. (a) 2-theta-omega (2θ-ω) scans close to (001) STO of all films. SMO:BSO and SMO films show clear SMO and BSO phases. (b) φ-scan of the (111) reflection of STO substrate and three films. All films show the epitaxial relationship between SMO and STO of [100]SMO//[110]STO or [010]SMO//[110]STO.

XRD. Supplementary Figure 1a shows a 2 theta-omega (2θ-ω) X-ray scan close to (001) STO of all films. All the (001) BSO peaks are clearly visible for the VAN films. Also, all the films show an *o*-SMO peak just below STO (001) peak, labelled as ‘S’, for strained SMO. When <110> SMO aligns with 2×<100> STO, it is compressed by the STO in-plane by ~-1.1% (see Supplementary Table 1) which leads to an out-of-plane tension in the SMO. The ‘S’ peak is very close to STO which also in agreement with RSMs (Fig. 4). More details on the evolution of the strain in the 100 nm VAN film are explored in Figure 4 and Supplementary Figure 2.

The 100 nm SMO plain and VAN films both show an additional peak labelled as ‘R’ to the right of the STO peak. This corresponds to there being a portion of relaxed SMO in the films. The ‘R’ peak would not appear in the RSMs of Figure 4 as the 2θ value is too high.

Supplementary Figure 1b shows φ-scans of the (111) reflection of STO substrate and SMO film to determine epitaxial relationship. For all films, four narrow STO (111) peaks, 90° in-plane spaced, are visible together with four *o*-SMO (111) reflections, located at 45° from each STO (111) reflection. This indicates that the in-plane axes of the film are 45° in-plane rotated respect to the (100) direction of the substrate. Therefore, all films have an epitaxial relationship of [100]SMO//[110]STO or [010]SMO//[110]STO. These results reveal that the SMO thin films are orthorhombic.[1–5]

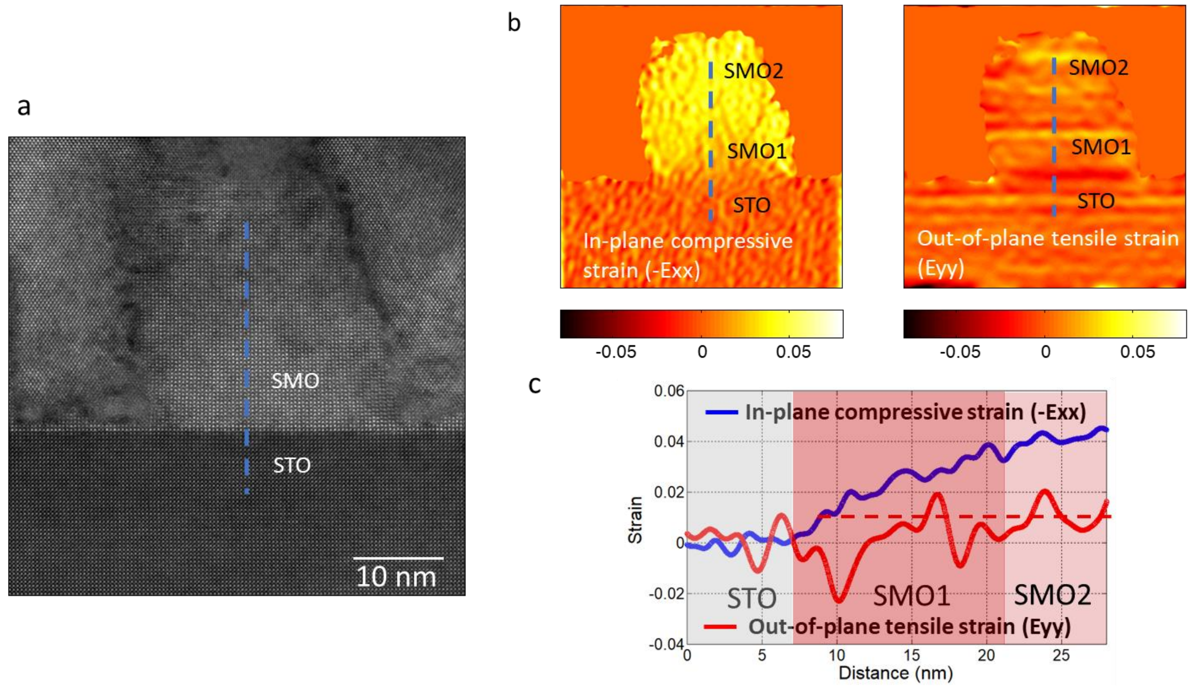

Supplementary Figure 2. Strain distribution in SMO in 100 nm VAN SMO:BSO films. **a** High-resolution STEM image showing the region of SMO used for the strain calculations. **b** In-plane compressive strain (-E_{xx}) map and out-of-plane tensile strain (E_{yy}) map, calculated from the image in Supplementary Figure 2a. The *o*-SMO2 is brighter indicative of higher in-plane compression. **c** Plot of the strain versus distance across the vertical line shown in Supplementary Figure 1a and b. The strain maps in Supplementary Figure 1b are calculated with respect to the STO lattice parameter (a = 3.905 Å). The horizontal dashed red line is an average of the strain from the solid red line. It shows ~1% out-of-plane tensile strain in the SMO with respect to the STO, or ~ 5.2% w.r.t. bulk SMO. The calculated maximum ~4% in-plane compressive strain (blue line) w.r.t. STO corresponds to ~5.0% w.r.t. bulk SMO was observed in *o*-SMO2. The approximate locations of the o-SMO1 and *o*-SMO2 regions are shown.

HR-TEM. Supplementary Figure 2 shows the strain distribution in the SMO film calculated using a high-resolution STEM image [6]. The noise in the strain map is due to the instability of the electron probe during STEM image acquisition. The noise level is higher (~1-2%) in the out-of-plane strain map (Supplementary Fig. 2b), as expected from the scanning probe instability. The SMO pillar has an out-of-plane tensile strain of ~1% on the average (indicated by horizontal dashed red line in Supplementary Fig. 2c) and an increasing compressive in-plane strain with film thickness up to ~4%. It is important to point out the strain maps (Supplementary Fig. 2b) and strain line-profile (Supplementary Fig. 2c) were calculated with respect to the STO lattice parameter (a = 3.905 Å). With respect to bulk SMO lattice parameters the ~1% out-of-plane tensile strain corresponds to ~5.2%, and the ~4% in-plane compressive strain corresponds to ~5.0%.

Although the out-of-plane tensile strain observed in the SMO is too noisy to accurately determine the strain level, the averaged level of ~1% w.r.t. STO is consistent with the 0-0.64% strain levels determined for the 100 nm VAN film shown in Supplementary Table 1. On the other hand, the in-plane compressive strain of up to ~4% w.r.t. STO, is larger than the 2.5%

determined from XRD (Supplementary Table 1). However, the TEM is looking only locally whereas X-ray averages over a much larger region of the film. Overall, the TEM strain analysis confirms the X-ray data of very large in-plane compressive strain and out-of-plane tensile strain induced in the 100 nm VAN films.

Supplementary Table 1. Comparison of four films grown in this study. Cell parameters, unit cell volume, strain and physical properties comparing SMO of different forms. We note that since SMO is rotated in-plane by 45° with respect to the [100] STO direction, then the lattice parameters of SMO closely match with the STO in-plane lattice parameters diagonally ($\sqrt{2} \times 3.905$ Å = 5.523 Å). We recall that the 45° in-plane rotation of SMO on STO is confirmed by the X-ray phi scan (Supplementary Fig. 1b). Hence, [110] SMO is parallel to the [100] STO. We include the SMO d_{110} values in Supplementary Table 1 as this shows the average lattice misfit which matches with 2 × <001> STO.

In the RSM of the 20 nm VAN film the (026) peak of SMO is not well separated from the (113) peak of STO. As result, the *b* lattice parameter is assumed to be very close to STO. The lattice parameters and strain levels in the 100 nm VAN film are highlighted because these are compared with the values calculated from STEM images in Supplementary Figure 2.

Film type		*a* (Å)	*b* (Å)	d_{110} (Å)	*c* (Å)	V (Å^3)	Measured strain (%) in SMO film *cf.* bulk SMO (bulk STO)				Physical properties
							a	***b***	$\boldsymbol{d_{110}}$	***c***	
					**Film**						
**100 nm SMO:BSO**	*o*-SMO1	5.505 ±0.005	5.553 ±0.005	7.819 ±0.005	7.810 ±0.002	240.7 ±0.1	2.7	-4.1	**-0.9 (0.01)**	**4.2 (0)**	FM+AFM/FE T_C = 90 K T_N = 60 K FE at RT
	o-SMO2	5.338 ±0.005	5.427 ±0.005	7.612 ±0.005	7.86 ±0.01	227.7 ±0.1	-0.4	-6.3	**-3.6 (-2.5)**	**4.9 (0.64)**	
**20 nm SMO:BSO**	*o*-SMO1	5.508 ±0.005	5.553 ±0.005	7.819 ±0.005	7.805 ±0.002	238.7 ±0.1	2.7	-4.1	-0.9	4.2	FM+AFM T_C = 70 K T_N = 60 K
**100 nm SMO**	*o*-SMO1	5.511 ±0.005	5.522 ±0.005	7.801 ±0.005	7.816 ±0.002	237.8±0.1	2.8	-4.7	-1.1	4.3	AFM T_N = 60 K
					**Bulk**						
							**Calc. strain (%) in bulk SMO required to match√2 x STO**				
**$SmMnO_3$** [7]		5.3601	5.7937	7.8929	7.4932	232.7	3.0	-4.7	-1.0	-	A-AFM T_N = 60 K PE
**√2 x $SrTiO_3$**		5.522	5.522	7.810	5.522	-	-	-	-	-	-
**$(Bi_{0.62},Sm_{0.38})_2O_3$** [8]		11.1017	11.1017		11.1017	-	-	-	-	-	-

Supplementary Table 2: DFT Calculation results. DFT calculations results estimating the Mn-O-Mn bond angles and Mn-O bond lengths in SMO, both out-of-plane (OOP) and in-plane (IP). The calculation shows drastic change in OOP bond angle and IP bond length. The OOP bond length and IP bond angle changes slightly which agrees with the proposed mechanism for enhanced Mn-Mn interaction.

Composition		OOP Angle (°)	OOP Length (Å)	IP Angle (°)	IP Length (Å)
**100 nm SMO:BSO**	*o*-SMO1	143.722	2.054605	146.9846	2.038627
	o-SMO2	147.3613	2.047493	147.0291	1.984555
**100 nm SMO**		143.9184	2.055033	147.148	2.033173
**SMO-Bulk**		143.5921	1.971997	146.2775	2.061429

Ferroelectric measurement – *P-E* hysteresis loops: The *P-E* loops were measured on three samples (Supplementary Fig. 3). The samples were the thick (100 nm) SMO:BSO VAN film, the thin (20 nm) SMO:BSO VAN film and the thick (100 nm) plain SMO film. Only the 100 nm SMO:BSO VAN film shows ferroelectricity at room temperature.

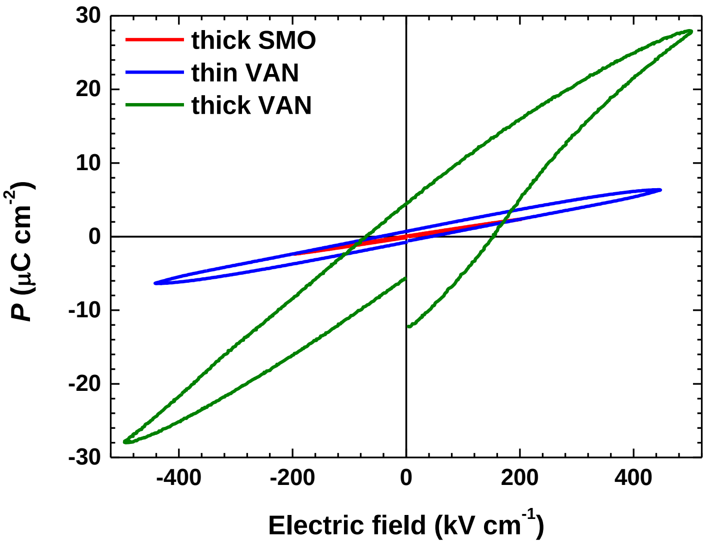

Supplementary Figure 3. *P-E* hysteresis measurements on three different films. Only the thick VAN film shows a clear FE loop.

Ferroelectric measurement - PUND measurements: PUND measurements were undertaken at RT to obtain more confirmative remanent (switching) polarization data. The PUND measurements were done with 3 different pulses, 1, 0.1 and 0.01 ms (Supplementary Fig. 4). The remanence polarizations were found to be slightly decreased with decreasing pulse time from 1 ms to 0.1 ms. For 0.01 ms remanence polarization value drops significantly due to instrument limitation (minimum pulse width is 50 μs). It is also possible that FE leakage, electrical parasitic effects from the 3D VAN structure, etc. can affect the polarization value.

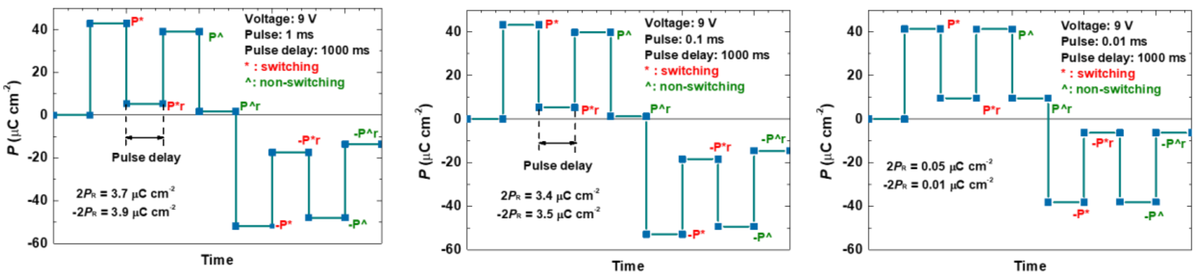

Supplementary Figure 4. PUND measurements for different pulse steps.

Ferroelectric measurement - PUND in PFM (nano-PUND): PUND measurements were also done in PFM mode, termed as nano-PUND (also termed AFM-PUND).[9,10] Having a small contact area, defined primarily by the tip, the nano-PUND measurement has low signal to noise ratio. A solid blunted Pt tip with diameter of the order of 200 nm was used, similar to the recent work be O. Kwon, *et al.* [10]

The local nano-PUND measurements were performed using a commercial Scanning Probe Microscope: Agilent 5500. The measurements were done by applying switching and non-switching voltages - via the back (substrate) electrode of the sample and a conductive AFM tip connected to a trans-impedance amplifier with the input at a virtual zero. In this method, the voltage was applied to the sample electrode (either deposited metallic electrode in the macroscopic case, or an AFM tip in the local-nanoscale case) and the current was measured. However, the triangular sweeps were applied with either positive and/or negative voltage, in pairs.

The first sweep (marked as 1 in Supplementary Figure 5a, the schematic diagram of the nano-PUND measurement, being adopted from O. Kwon, *et al.*[10]), induces switching and registers the total current of switching, leakage and linear dielectricity, while the second (of the same polarity) sweep (marked as 2 in the Supplementary Fig. 5a) does not involve switching (due to the film been switched already). At the nano-scale area, precautions were taken to avoid domain growth. To do that, background pooling (of a larger region) and pre-poling were made before every spectroscopic sweep.[10] By subtracting the current of the second sweep from the first one, the absolute switching current was measured. This difference in switching current, ΔI, is directly connected to the switching charge transferred during the measurement time t. The polarization was then obtained from the time integrated charge transfer divided by the electrode/tip contact area: $P = \int I\delta t$ /Area. A low frequency was used (sub Hz) in order to allow small currents to be measured, employing a feedback via a resistor of the order of 10 GΩ. This enabled a maximum current of 1 nA to be measured using a maximum 10 V bias.

The nano-PUND measurement result is shown in Supplementary Figure 5b. The pulse time was 0.5 sec and there was no delay for the next pulse. A clear split peak was observed in the 1st sweep which is absent in the second sweep both in the positive and negative directions. Supplementary Figure 5c shows the corresponding switching current. By using the same method, ferroelectric loops for two different frequencies and voltages are also obtained (Supplementary Fig. 5d).[10] Frequency clearly does not affect the polarization as long as it is above the switching voltage. The nano-PUND method enables the switching current to be distinguished from the non-switching current. This enables the measurement of the polarization due to ferroelectricity as a function of voltage. Since the low voltage loop (5V) is tiny and does not switch but the 10V loop is large and switches completely, this clearly proves ferroelectricity.

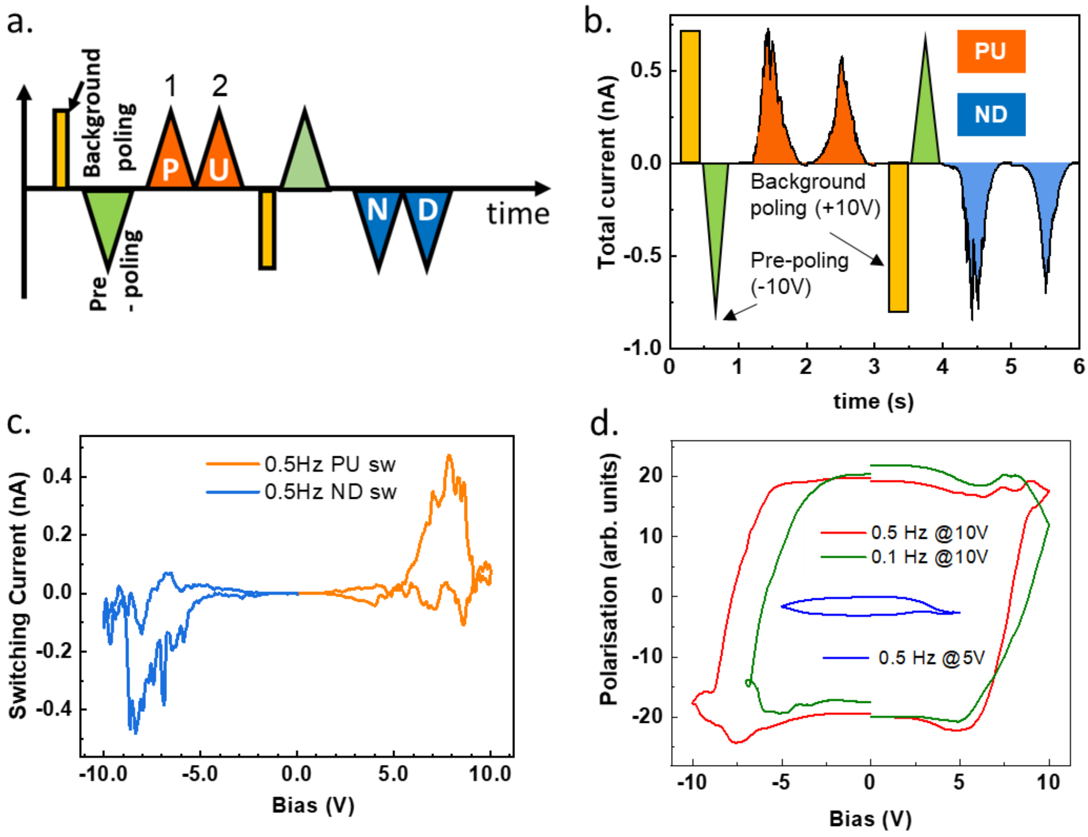

Supplementary Figure 5. Nano-PUND measurements by PFM. **a** Schematic diagram of the nano-PUND sequence (similar to ref 10). **b** The measured nano-PUND switching of VAN films. **c** The ferroelectric switching current acquired by the nano-PUND waveform. **d** The *P-E* hysteresis loop measured by the nano-PUND method for two different frequencies (0.1 Hz and 0.5 Hz) and voltages (10 V and 5 V). Polarization data for the 5 V data is magnified by a factor of 10.

SHG - Theoretical Modeling of SHG Polarimetry. The theoretical modeling of experimental SHG polar plots was performed by using the similar method described in Garten *et al.*[11] The third rank second order nonlinear optical SHG d tensor (d_{ijk}) can be calculated with linear orthogonal transformation (LOT) and Neumann's Principle.[12] The third rank SHG d tensor then can be simplified with Voigt notation (d_{ij}^{Voigt}) to 3×6 matrix, by the symmetry consideration. When c-axis is out of plane, the point group *4* in the 2 cases of in-plane orientations (ψ= 0°, 90°) have the same form of SHG d tensor in the sample axis:

$$d_{ij}^{Voigt} = \begin{pmatrix} 0 & 0 & 0 & 0 & d_{15} & 0 \\ 0 & 0 & 0 & d_{15} & 0 & 0 \\ d_{31} & d_{31} & d_{33} & 0 & 0 & 0 \end{pmatrix}$$

The expression for second harmonic electric field in the lab axis can be calculated by performing axis transformation and matrix multiplication:

$$E_{i,x}^{2\omega}(\theta = 45°, \psi = 0°) \propto \frac{1}{4}[2\sqrt{2}d_{15}R_p^2\cos^2\varphi] - \sqrt{2}[d_{31}(R_p^2\cos^2\varphi + 2R_s^2\sin^2\varphi) + d_{33}R_p^2\cos^2\varphi]$$

$$E_{i,y}^{2\omega}(\theta = 45°, \psi = 0°) \propto \sqrt{2}R_pR_sd_{15}\cos\varphi\sin\varphi$$

$$E_{i,x}^{2\omega}(\theta = 45°, \psi = 90°) \propto -\frac{1}{4}[2\sqrt{2}d_{15}R_p^2\cos^2\varphi] + \sqrt{2}[d_{31}(R_p^2\cos^2\varphi + 2R_s^2\sin^2\varphi) + d_{33}R_p^2\cos^2\varphi]$$

$$E_{i,y}^{2\omega}(\theta = 45°, \psi = 90°) \propto \sqrt{2}R_pR_sd_{15}\cos\varphi\sin\varphi$$

Where R_p and R_s are Fresnel's reflection coefficient determined from refractive index of the film. When modeling, the refractive index of the film was set as fitting parameter, constrained from 2 to 3, which are typical values for insulating oxide. The SHG intensity is given by:

$$I_x^{2\omega} \propto |E_{i,x}^{2\omega}|^2 \text{ and } I_y^{2\omega} \propto |E_{i,y}^{2\omega}|^2$$

Where $I_x^{2\omega}$ and $I_y^{2\omega}$ represent SHG intensity of p- and s-wave, respectively. As shown above, since $E_{i,x}^{2\omega}(\theta = 45°, \psi = 0°) = -E_{i,x}^{2\omega}(\theta = 45°, \psi = 90°)$ and $E_{i,y}^{2\omega}(\theta = 45°, \psi = 0°) = E_{i,y}^{2\omega}(\theta = 45°, \psi = 90°)$, and the SHG intensity is proportional to square of electric field, the SHG intensities are identical, regardless of in-plane orientation angle ψ.

SHG - Temperature dependent measurement. The temperature dependent SHG measurement was done over the temperature range of room temperature to 623K (350° C). A change in slope showed the onset of saturation of SHG to be below ~360-370 K in both cooling and heating measurements. The origin of the second hump in SHG observed near ~500 K is currently of unknown origin.

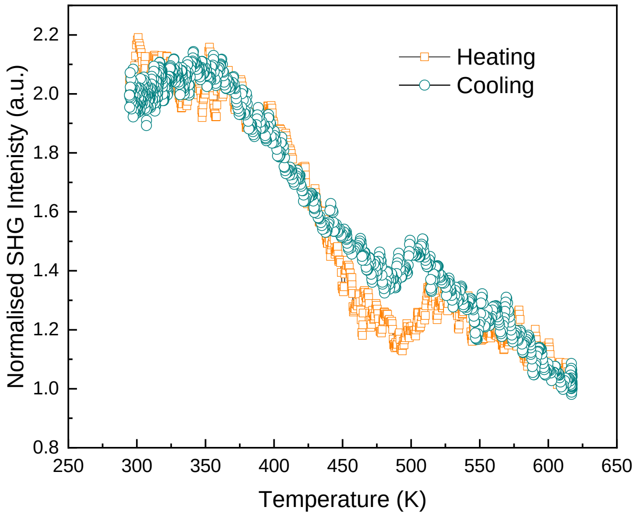

Supplementary Figure 6: Temperature dependent SHG of 100 nm thick VAN SMO:BSO film showing the onset of long range polar order at ~ 360-370 K.

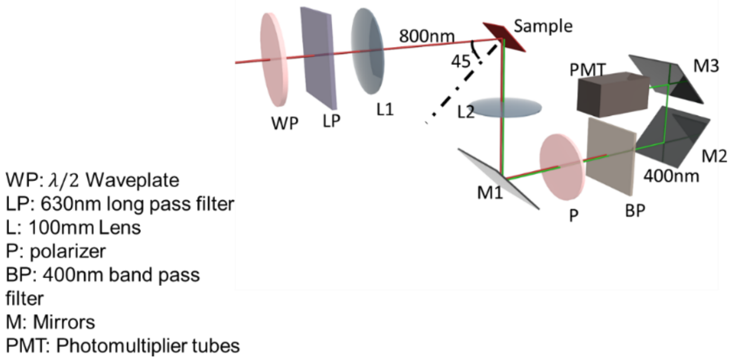

Supplementary Figure 7. SHG measurement schematic setup

Supplementary References:

1. Marti, X. *et al.* Emergence of ferromagnetism in antiferromagnetic $TbMnO_3$ by epitaxial strain. *Appl. Phys. Lett.* **96**, 2010–2012 (2010).

2. Cui, Y. M., Tian, Y. F., Shan, A. X., Chen, C. P. & Wang, R. M. Magnetic anisotropy and anomalous transitions in $TbMnO_3$ thin films. *Appl. Phys. Lett.* **101**, 5 (2012).

3. Venkatesan, S., Daumont, C., Kooi, B. J., Noheda, B. & De Hosson, J. T. M. Nanoscale domain evolution in thin films of multiferroic $TbMnO_3$. *Phys. Rev. B - Condens. Matter Mater. Phys.* **80**, 1–7 (2009).

4. Li, X. *et al.* Novel multiferroicity in $GdMnO_3$ thin films with self-assembled nano-twinned domains. *Sci. Rep.* **4**, 7019 (2014).

5. Daumont, C. J. M. *et al.* Epitaxial $TbMnO_3$ thin films on $SrTiO_3$ substrates: A structural study. *J. Phys. Condens. Matter* **21**, (2009).

6. Hÿtch, M. J., Snoeck, E. & Kilaas, R. Quantitative measurement of displacement and strain fields from HREM micrographs. *Ultramicroscopy* **74**, 131–146 (1998).

7. O'Flynn, D., Tomy, C. V., Lees, M. R., Daoud-Aladine, A. & Balakrishnan, G. Multiferroic properties and magnetic structure of $Sm_{1-x}Y_xMnO_3$. *Phys. Rev. B* **83**, 174426 (2011).

8. Watanabe, A. Preparation of a New Phase Having a Cation-Ordered C-Type Rare-Earth Oxide Related Structure in the Systems Bi_2O_3-Ln_2O_3 (Ln = Sm, Eu, Gd, Tb, and Dy). *J. Solid State Chem.* **120**, 32–37 (1995).

9. Martin, S., Baboux, N., Albertini, D. & Gautier, B. A new technique based on current measurement for nanoscale ferroelectricity assessment: Nano-positive up negative down. *Rev. Sci. Instrum.* **88**, (2017).

10. Kwon, O. *et al.* Direct Probing of Polarization Charge at Nanoscale Level. *Adv. Mater.* **30**, 1–8 (2018).

11. Garten, L. M. *et al.* Relaxor Ferroelectric Behavior in Barium Strontium Titanate. *J. Am. Ceram. Soc.* **99**, 1645–1650 (2016).

12. *F. E. Neumann (1985), Vorlesungen über die Theorie der Elastizität der festen Körper und des Lichtäthers*. (Leipzig, B. G. Teubner-Verlag).
